# Long-term prognostic significance of history of cancer and atrial fibrillation in coronary artery disease

**DOI:** 10.1016/j.ijcha.2023.101277

**Published:** 2023-10-11

**Authors:** Kotaro Nochioka, Takashi Shiroto, Hideka Hayashi, Takumi Inoue, Kazuma Oyama, Kai Susukita, Hiroyuki Takahama, Jun Takahashi, Hiroaki Shimokawa, Satoshi Yasuda

**Affiliations:** a Department of Cardiovascular Medicine, Tohoku University Graduate School of Medicine, Japan; b Clinical Research, Innovation and Education Center, Tohoku University Hospital, Japan; c International University of Health and Welfare, Narita, Japan

**Keywords:** Coronary artery disease, Cancer, Atrial fibrillation, Anticoagulant, Antiplatelet

## Abstract

**Background:**

Limited data exist on the prognostic significance of a history of cancer and atrial fibrillation (AF) in patients with coronary artery disease (CAD). This study aimed to evaluate the associations among a history of cancer, AF, and long-term prognosis in patients with CAD.

**Methods:**

We studied 3,233 patients with CAD (69 ± 11 years; women, 23%) in a multicenter hospital-based cohort study, the CHART-2 and related a history of cancer and AF to cardiovascular outcomes with a median follow-up of 10.8 years.

**Results:**

Of the 3,233 patients enrolled, 10.7% and 11.2% had a history of cancer and AF, respectively, while 2.8% had both. Patients with AF and a history of cancer were characterized by older age, male sex, and higher BNP levels. Anticoagulant use with warfarin or direct oral anticoagulants increased from 43% at baseline to 56% at 10 years in patients with CAD with AF and no history of cancer and increased from 49% to 83% in those with both. Patients with CAD with both comorbidities had a higher risk of a composite outcome including stroke, thrombosis, and major bleeding (Hazard Ratio [HR_adjusted_], 2.26; 1.50–3.40, P < 0.001). Furthermore, patients with both comorbidities had a higher risk of all-cause death (1.55; 95% confidence interval [CI] 1.12–2.12, P = 0.007) including cancer death (2.62; 1.51–4.54, P = 0.001), and new-onset heart failure (HF) requiring hospitalization (2.47; 1.54–3.96, P < 0.001).

**Conclusions:**

These results demonstrate that CAD patients with a history of cancer and AF have an increased risk of composite outcomes, including stroke, systemic thrombosis, major bleeding, all-cause death, cancer-related death, and new-onset HF.

## Highlights

• Coexisting history of cancer and AF increased the risk for clinical outcomes in CAD.
• Anticoagulant use increased for 10 years in patients with history of cancer and AF.
• These results highlight prognostic significance of history of cancer and AF in CAD.

## Graphical abstract

**Figure:**
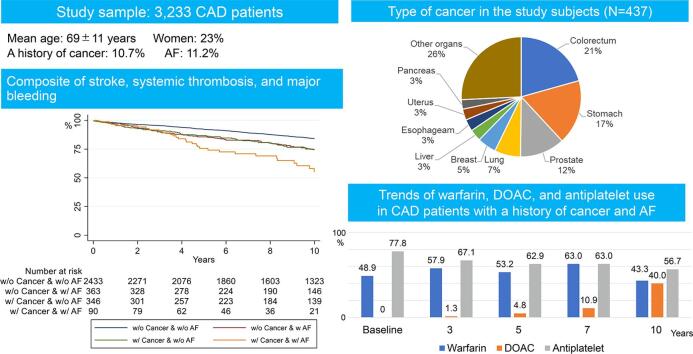

## 1 Introduction

Improvements in life expectancy have transformed cancer into a chronic condition, posing challenges in the management of comorbidities, especially when it comes to cardiovascular diseases, including coronary artery disease (CAD) or atrial fibrillation (AF) [1]. Treatment-related adverse events and drug-drug interactions often influence the therapeutic approach for patients with cancer and cardiovascular disease. Tumor cells and blood clotting compromise this complex crosstalk and induce hemostasis abnormalities [2].

Cancer and cardiovascular disease share common risk factors including age, obesity, smoking, alcohol, and chronic inflammation [3]. Prior studies have reported that patients with cancer are at increased risk of developing AF and CAD [4], [5]. A recent *meta*-analysis showed that patients with cancer had a 1.5-fold increased risk of developing AF compared to those without cancer [4]. Additionally, cancer survivors have been found to have an increased risk of developing CAD [6], [7]. Moreover, a previous study found a bidirectional association between the presence of CAD and an increased risk of death in patients with cancer [8]. Despite the emerging evidence implying a close relationship among cancer, AF, and CAD, limited evidence exists regarding long-term predictive value of a history of cancer and AF in patients with CAD. Furthermore, there is no consensus regarding the use of antithrombotic treatments in patients with both cardiovascular diseases and cancer.

In the present study, we aimed to evaluate the prognostic significance of a history of cancer and AF for mortality, stroke, thrombosis, and bleeding in CAD using the dataset from a hospital-based prospective cohort named the Chronic Heart Failure Analysis and Registry in the Tohoku District-2 (CHART-2) study [9], [10].

## 2 Methods

### 2.1 Study setting

CHART-2 (NCT00418041) is a hospital-based prospective observational study from six Japanese prefectures (Aomori, Akita, Iwate, Miyagi, Yamagata, and Fukushima) [9]. The design and methods have been previously described in detail [9]. Briefly, from October 2006 and March 2010, we enrolled consecutive patients older than 20 years with significant CAD and those in Stage B (structural heart disease but without signs or symptoms of HF), Stage C (structural heart disease with earlier or current symptoms of HF), and Stage D enumerated by the American College of Cardiology Foundation/ American Heart Association guidelines [9]. Stage B was defined by echocardiographic and clinical findings as follows; enlarged left ventricular diameter (LVDD ≥ 55 mm), reduced left ventricular ejection fraction (LVEF ≤ 50%), thickened interventricular septum (>12 mm) and/or thickened left ventricular posterior wall (>12 mm), valvular heart disease, wall motion abnormalities, congenital abnormalities, and previous cardiac surgery (such as coronary artery bypass grafting). All information, including medical history, laboratory data, and echocardiography data, were recorded in a computer database at the time of enrollment. Annual follow-ups were conducted by clinical research coordinators by reviewing medical records, surveys, and telephone interviews.

Of 10,219 patients (mean age 68.3 ± 12.1 years, women 30.4%, history of cancer 13.7%, and AF 32.2%), we excluded patients with HF (n = 4,918; mean age 69.0 ± 12.3 years, women 31.9%, history of cancer 13.8%, AF 41.1%, LVEF < 45% 22.5%, and median brain natriuretic peptide (BNP)(interquartile range [IQR] 104 [41–239] pg/mL), and Stage B patients without CAD (n = 2,068; mean age 66.3 ± 13.6 years, women 38.4%, history of cancer 14.0%, AF 39.5%, valvular heart disease 37%, and hypertensive heart disease 21%). The final analysis included 3,233 patients with CAD in the present study.

### 2.2 Ethical approval

This study conformed to the principles of the Declaration of Helsinki. The study protocol was approved by the institutional review board of each participating center, and all participants provided written informed consent.

### 2.3 History of cancer

A history of cancer was collected at baseline assessment as part of the medical history. Patients were considered to have a “history of cancer” if their past medical history included the following terms: intracranial neoplasm; brain cancer; leukemia; lymphoma; lung cancer; stomach cancer; esophageal cancer; colorectal cancer; liver cancer; breast cancer; thyroid cancer; endometrial, ovarian cancer; renal, bladder, or urinary tract cancers; or metastatic cancer. Baseline medications were reviewed to identify patients undergoing cancer treatment with hormonal or chemotherapeutic agents. These patients were defined as actively treated for cancer and were excluded from the group with a history of cancer. No additional information on the cancer stage or treatment was obtained.

### 2.4 Outcome

The primary outcome was a composite of stroke, thrombosis, and major bleeding events. The secondary outcomes included all-cause death, cardiovascular death, cancer-related death, stroke, and new-onset HF requiring hospitalization. Stroke was defined as the loss of neurological function caused by an ischemic or hemorrhagic event with residual symptoms for at least 24 h after onset on imaging (computed tomography [CT] or magnetic resonance imaging [MRI]). Major bleeding was defined according to the International Society on Thrombosis and Hemostasis criteria [11]. All events were adjudicated by consensus of three independent physicians who were members of the Tohoku Heart Failure Association. They reviewed the case reports, death certificates, medical records, and summaries provided by the investigators [9].

### 2.5 Statistical methods

Clinical characteristics were described based on the history of cancer and AF. We then evaluated the association between a history of cancer, AF, and long-term outcomes. Based on a literature search [5], [12], [13], we built Cox hazard models considering the following variables as potential confounders: age, sex, LVEF, systolic blood pressure, heart rate, body mass index, smoking status, diabetes, BNP level, and estimated glomerular filtration rate (eGFR). Subsequently, we tested for inclusion in the regression models by stepwise selection, with a significance level of P < 0.05. A two-sided P-value of < 0.05 was considered to be statistically significant. All analyses were performed using STATA 17 software (College Station, TX, USA).

## 3 Results

### 3.1 Baseline characteristics

Of the 3,233 study participants, the mean age was 69 ± 11 years, 23% were women, 10.7% and 11.2% had a history of cancer and AF, respectively, and 2.8% had both. A history of stroke was observed in 19% of the participants. Table 1 shows the comparisons among the four groups: CAD patients with and without AF and those with and without a history of cancer. Among the four groups, patients with AF and a history of cancer were the oldest, most frequently male, and most likely to have the highest BNP levels. In addition, this group had the highest prevalence of a history of CABG. Among the patients with a history of cancer (n = 437), 21% had colorectal cancer, 17% had stomach cancer, 12% had prostate cancer, 7% had lung cancer, 5% had breast cancer, 3% had liver cancer, 3% had esophageal cancer, 3% had cervical cancer, 3% had pancreatic cancer, and 26% had other cancers.

**Table 1:** Baseline characteristics stratified by history of cancer and AF.

	Overall n = 3,233	w/o Hx cancer and w/o AF n = 2,433	w/o Hx cancer and w/ AF n = 363	w/ Hx cancer and w/o AF n = 347	w/ Hx cancer and w/ AF n = 90	P-value
Age, yrs	69 ± 11	67 ± 11	72 ± 10	72 ± 8	75 ± 7	<0.001
Women, n (%)	736 (23%)	575 (24%)	70 (19%)	72 (21%)	19 (21%)	0.21
Systolic BP, mmHg	130 ± 17	130 ± 17	129 ± 18	131 ± 17	130 ± 19	0.49
Diastolic BP, mmHg	74 ± 11	75 ± 11	74 ± 12	74 ± 11	73 ± 12	0.51
Heart rate, bpm	69 ± 13	69 ± 12	71 ± 13	69 ± 12	75 ± 16	<0.001
Body mass index	24.3 ± 3.2	24.4 ± 3.2	24.2 ± 3.2	23.9 ± 3.3	23.5 ± 3.7	0.002
Hypertension, n (%)	2961 (92%)	2219 (91%)	342 (94%)	316 (91%)	84 (93%)	0.24
Diabetes, n (%)	1373 (43%)	1039 (43%)	150 (41%)	154 (44%)	30 (33%)	0.28
Dyslipidemia, n (%)	2904 (90%)	2205 (91%)	305 (84%)	320 (92%)	74 (82%)	<0.001
Previous myocardial infarction, n (%)	1519 (47%)	1205 (50%)	139 (38%)	133 (38%)	42 (47%)	<0.001
History of stroke, n (%)	628 (19%)	431 (18%)	109 (30%)	64 (18%)	24 (27%)	<0.001
PCI, n (%)	2178 (67%)	1708 (70%)	204 (56%)	213 (61%)	53 (59%)	<0.001
CABG, n (%)	366 (11%)	267 (11%)	39 (11%)	45 (13%)	15 (17%)	0.27
PMI, n (%)	65 (2%)	27 (1%)	28 (8%)	6 (2%)	4 (4%)	<0.001
Valve surgery, n (%)	27 (1%)	15 (1%)	8 (2%)	1 (0.3%)	3 (3%)	<0.001
Hemoglobin, g/dL	13.6 ± 1.6	13.6 ± 1.6	13.6 ± 1.6	13.2 ± 1.7	12.9 ± 1.8	<0.001
eGFR, mL/min/1.73 m^2^	66.2 ± 18.5	67.5 ± 18.6	62.8 ± 17.3	62.2 ± 17.1	59.8 ± 19.8	<0.001
LVEF, %	64.2 ± 11.3	64.2 ± 11.3	63.7 ± 11.7	64.4 ± 10.7	63.5 ± 11.2	0.78
LVDD, mm	48.6 ± 6.5	48.6 ± 6.5	49.3 ± 6.5	48.0 ± 6.4	47.9 ± 7.2	0.07
BNP	39 [19, 90]	35 [17, 72]	89 [43, 174]	43 [19, 91]	118 [68, 206]	<0.001
PT-INR*	1.8 [1.5, 2.1]	1.8 [1.5, 2.1]	1.8 [1.5, 2.1]	1.7 [1.6, 2.3]	1.7 [1.5, 2.1]	0.86
Beta blocker, n (%)	1066 (33%)	791 (33%)	134 (37%)	108 (31%)	33 (37%)	0.27
RASI, n (%)	1920 (59%)	1462 (60%)	223 (61%)	186 (54%)	49 (54%)	0.08
Ca blocker, n (%)	1858 (58%)	1383 (57%)	229 (63%)	191 (55%)	55 (61%)	0.09
Diuretics, n (%)	386 (12%)	263 (11%)	67 (19%)	35 (10%)	21 (23%)	<0.001
Statin, n (%)	1928 (60%)	1544 (64%)	163 (45%)	185 (53%)	36 (40%)	<0.001
Antiplatelets, n (%)	2817 (87%)	2153 (89%)	295 (81%)	299 (86%)	70 (78%)	<0.001
Anticoagulants, n (%)	442 (14%)	213 (9%)	156 (43%)	29 (8%)	44 (49%)	<0.001

*Abbreviations:* BNP, brain natriuretic peptide; BP, blood pressure; CABG, coronary artery bypass grafting; eGFR, estimated glomerular filtration rate; LVEF, left ventricular ejection fraction; LVDD, left ventricular end-diameter; PCI, Percutaneous coronary intervention; PT-INR, Prothrombin time and international normalized ratio; PMI, pacemaker implantation; RASI, renin-angiotensin system inhibitor.

*PT-INR was obtained in 213, 156, 29, and 44 cases with warfarin in w/o Hx cancer and w/o AF, w/o Hx cancer and w/ AF, Hx cancer and w/o AF, and w/ Hx cancer and w/ AF, respectively.

### 3.2 Trends in antithrombotic and antiplatelet therapy

**Fig. 1:**
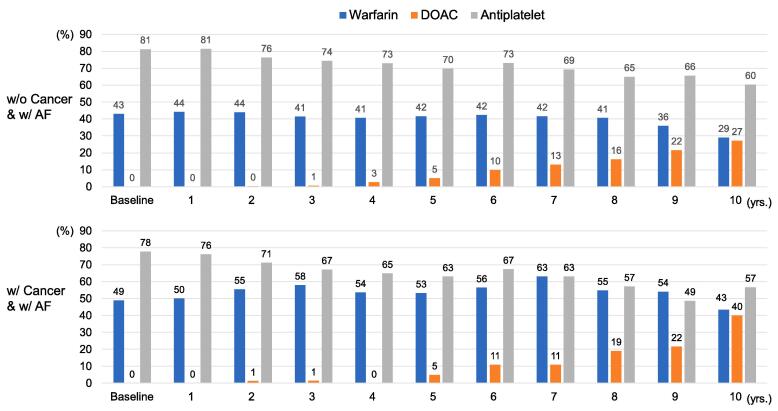
Ten-year trends in the use of warfarin, direct oral anticoagulant (DOAC), and antiplatelet in patients with CAD but without cancer and AF (upper) and those with cancer and with AF (lower).

At baseline (enrollment), 14% and 87% of all participants received anticoagulant (warfarin) and antiplatelet therapy, respectively, and the median prothrombin time and international normalized ratio (PT-INR) level was 1.8 (IQR, 1.5–2.1). At baseline, anticoagulant and antiplatelet therapies were administered to 43% and 81% of patients with AF without a history of cancer, and 49% and 78% of those with a history of cancer, respectively (Fig. 1). At 10 years, anticoagulant and antiplatelet therapies were received to 56% (direct oral anticoagulant; DOAC, 27%) and 60% of patients with AF without a history of cancer and 83% (DOAC, 40%) and 57% of those with a history of cancer, respectively (Fig. 1). Antiplatelet use decreased from 81.3% at baseline to 60.4% at 10 years in patients with CAD and AF who had no history of cancer and decreased from 77.8% to 56.7% in those with AF and a history of cancer.

### 3.3 Association of history of cancer and AF with clinical outcomes in CAD patients

**Fig. 2:**
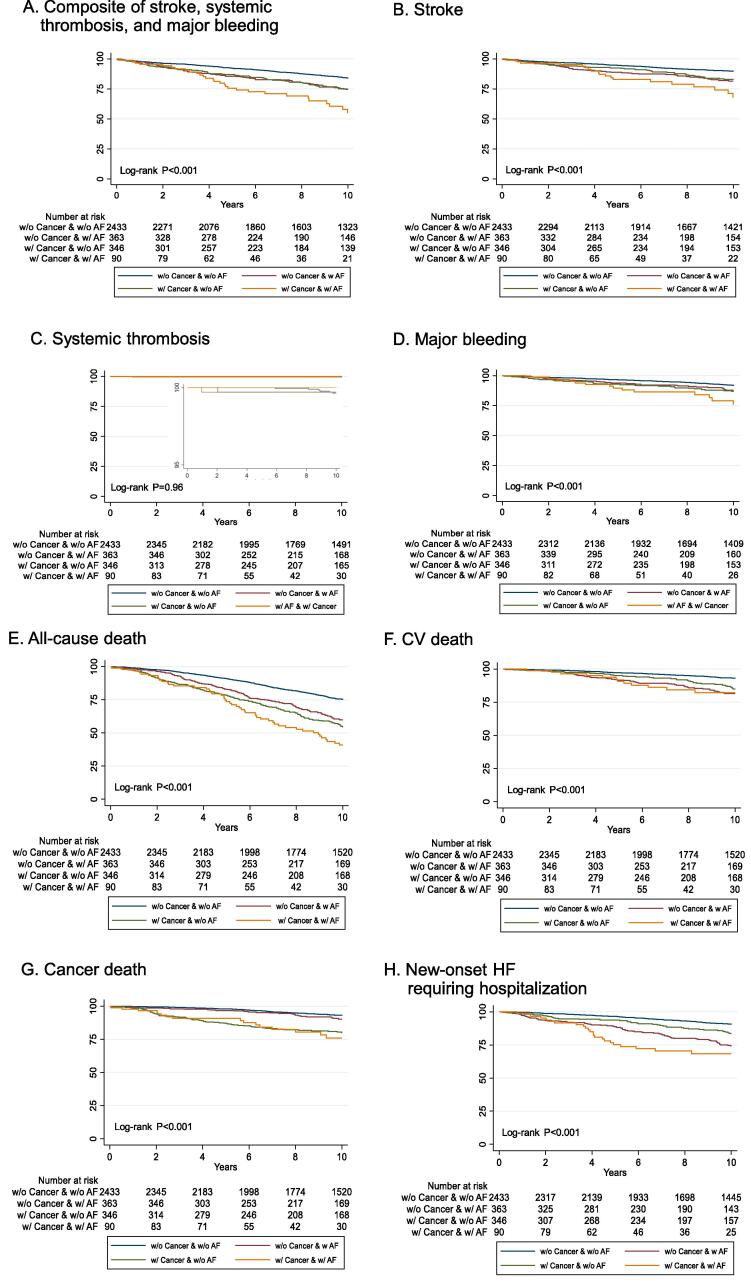
Kaplan–Meier curves for (A) composite of stroke, systemic thrombosis, and major bleeding, (B) stroke, (C) systemic thrombosis, and (D) major bleeding. (E) all-cause death, (F) cardiovascular (CV) death, (G) cancer death, and (H) new-onset HF requiring hospitalization,

Overall, patients with CAD and AF had higher clinical event rates (Fig. 2**A-H**). Patients with a history of cancer and AF had the highest event rates for the composite of stroke, systemic thrombosis, and major bleeding (Fig. 2**A-C**). Notably, patients with a history of cancer and AF had the highest major bleeding event rate among the four groups, even as antiplatelet use decreased over the course of 10 years (Fig. 1 **and** Fig. 2**D**). Furthermore, patients with a history of cancer and AF had the highest event rates of all-cause death (Fig. 2**E**) and new-onset HF requiring hospitalization (Fig. 2**H**). Importantly, patients with AF and no history of cancer had similar event rates of cancer-related death as those without AF and no history of cancer (Fig. 2**G**). After adjusting for age, sex, systolic blood pressure, heart rate, body mass index, history of stroke, smoking status, hypertension, diabetes mellitus, hemoglobin level, and eGFR, CAD patients with AF and a history of cancer had an increased risk for the composite of stroke, thrombosis, and major bleeding (adjusted hazard ratio, 2.26; 1.50–3.40, P < 0.001). (Table 2). Furthermore, the patients with AF and a history of cancer had a higher risk of all-cause death (1.55; 95% confidence interval 1.12–2.12, P = 0.007) including cancer death (2.62; 1.51–4.54, P = 0.001), and new-onset HF requiring hospitalization (2.47; 1.54–3.96, P < 0.001).

**Table 2:** Outcomes at 10 years.

	Composite of stroke, thrombosis, and major bleeding			All-cause death			Cancer death		
	No. (%)	HR_adjusted_ (95%CI)	P value	No. (%)	HR_adjusted_ (95%CI)	P-value	No. (%)	HR_adjusted_ (95%CI)	P-value
w/o Hx cancer and w/o AF (n = 2436)	328 (13.5)	(Reference)		555 (22.8)	(Reference)		137 (5.6)	(Reference)	
w/o Hx cancer and w/ AF (n = 361)	75 (20.8)	1.54 (1.18–2.00)	0.002	133 (36.8)	1.28 (1.04–1.56)	0.019	26 (7.2)	1.24 (0.80–1.92)	0.333
w/ Hx cancer and w/o AF (n = 341)	71 (20.8)	1.40 (1.06–1.84)	0.018	151 (44.3)	1.40 (1.15–1.69)	0.001	61 (17.9)	2.45 (1.77–3.40)	<0.001
w/ Hx cancer and w/ AF (n = 88)	29 (33.0)	2.26 (1.50–3.40)	<0.001	49 (55.7)	1.55 (1.12–2.12)	0.007	16 (18.2)	2.62 (1.51–4.54)	0.001

	New-onset heart failure requiring hospitalization			Stroke			Cardiovascular death		
	No. (%)	HR_adjusted_ (95%CI)	P-value	No. (%)	HR_adjusted_ (95%CI)	P-value	No. (%)	HR_adjusted_ (95%CI)	P-value
w/o Hx cancer and w/o AF (n = 2436)	190 (7.8)	(Reference)		215 (8.8)	(Reference)		140 (5.7)	(Reference)	
w/o Hx cancer and w/ AF (n = 361)	74 (20.5)	2.53 (1.90–3.37)	<0.001	55 (15.2)	1.67 (1.22–2.29)	0.001	54 (15.0)	1.92 (1.36–2.72)	<0.001
w/ Hx cancer and w/o AF (n = 341)	43 (12.6)	1.26 (0.89–1.80)	0.19	46 (13.5)	1.37 (0.97–1.93)	0.07	38 (11.1)	1.36 (0.92–2.00)	0.12
w/ Hx cancer and w/ AF (n = 88)	23 (26.1)	2.47 (1.54–3.96)	<0.001	19 (21.6)	2.29 (1.39–3.75)	0.001	12 (13.6)	1.53 (0.87–3.05)	0.13

Hazard ratios were adjusted for age, sex, systolic blood pressure, heart rate, body mass index, history of stroke, smoking status, hypertension, diabetes mellitus, hemoglobin level, and eGFR.

Abbreviations: AF, atrial fibrillation; CI, confidence interval; HR, hazard ratio.

## 4 Discussion

To the best of our knowledge, the present study is one of the largest to evaluate the prognostic significance of a history of cancer and AF in patients with CAD, providing important insights into the history of cancer as an important prognostic predictor. We report two novel findings; first, a coexisting history of cancer and AF increases the risk of all-cause death, cancer death, new-onset HF requiring hospitalization, stroke, and the composite of stroke, systemic thrombosis, and major bleeding, even after adjusting for potential confounders in patients with CAD. Second, in patients with CAD and AF, anticoagulant use increased over 10 years; however, event rates were higher in patients with a history of cancer and AF. These 10-year follow-up results highlight the prognostic significance of a history of cancer and AF in patients with CAD.

To date, most studies highlight a higher prevalence of AF in patients with cancer [14], [15]. A Danish nationwide cohort study reported that the incidence of AF was increased in all subtypes of cancer, showing the incidence of AF as 17.4/1,000 person-years in overall cancer as compared with 3.7/1,000 person-years in patients without cancer [15]. In contrast, some studies implied that AF itself may predict an increased risk of cancer diagnosis [16], [17]. Cancer and CAD also share overlapping risk factors, including but not limited to age, smoking, obesity, and diabetes [18]. Previous basic and clinical studies showed that myocardial infarction accelerate breast cancer growth and increase cancer-specific death [19], [20]. A report from Korea revealed an increased risk of de novo malignancy after percutaneous coronary intervention (PCI) compared to age- and sex-matched control patients who had not undergone PCI [8]. Of note, the study showed that the risk for lung and hematologic cancers was higher than that for other types of cancer [7]. Conversely, in patients with established cancer, the treatment for malignancy may have adverse effects on the genesis and progression of CAD [13]. Previous studies showed that radiation increased the risk for CAD [6], [7]. By demonstrating the prognostic importance of a history of cancer in patients with CAD, we now extend the prognostic relevance of a history of cancer and AF to patients with CAD. Notably, patients with CAD with a history of cancer and AF had the highest event rate for HF hospitalization.

Furthermore, there is limited evidence on the tolerability and efficacy of anticoagulants for bleeding, stroke, and/or systemic embolism in patients with cancer and AF. The present CHART-2 study demonstrated that in patients with CAD who had a history of cancer and AF, anticoagulant use increased over 10 years, with a growing preference for DOACs, although event rates remained higher. A recent Danish population-based cohort study found similar rates of thromboembolic events and bleeding in patients with and without cancer with AF compared to warfarin or DOAC therapy [21]. However, data supporting the preferential use of DOACs remain limited. It should also be mentioned that there was a robust trend of decreasing antiplatelet use in patients with CAD and AF over 10 years. This trend may be related to the guideline recommendation of oral anticoagulant monotherapy without antiplatelet therapy in stable CAD patients with AF [22], [23], [24]. Cancer is not a single entity and tumor behavior could differ among cancer types with different treatment modalities. It is possible that cancer type and tumor-specific factors could alter thrombotic risk in patients with cancer.

### 4.1 Study limitations

The present study has several limitations. First, as all study participants were Japanese, external validation of our findings is needed in other populations. Second, given CHART-2 was launched in 2006, anticoagulant therapy was administered along with warfarin at baseline in all cases. Third, we did not consider adherence, discontinuation, changes, or crossover of antiplatelet therapy in the present study. However, the PT-INR levels were within the therapeutic range throughout the study, suggesting acceptable adherence levels in the present population. Finally, the relatively small sample size of patients with a history of cancer and AF (n = 90) thwarted controlling the distributions of observed characteristics to be similar across the four groups, reducing confounding factors (e.g., propensity score matching).

## 5 Conclusions

The present study demonstrated that patients with CAD who had a history of cancer and AF have increased risks of a composite of stroke, systemic thrombosis, and major bleeding, all-cause death, cancer death, new-onset HF, warranting further investigation of optimal anticoagulation strategies in patients with CAD with both comorbidities.

## Funding

This study was supported in part by Daiichi Sankyo Co., Ltd.

## Declaration of Competing Interest

The authors declare the following financial interests/personal relationships which may be considered as potential competing interests: Kotaro Nochioka reports financial support was provided in part by Daiichi Sankyo Co., Ltd.
